# 

**Published:** 1903-08-15

**Authors:** 


### CONTENTS.

Porcelain as a Filling Material, its Qualities and Manipulation, - - *By Henry C. Raymond, D.D.S.* 379

Matrices, their Uses and Retention, *By Louis P. Hall, D.D.S.* 399

Dental Germicides, - *By E. Mawhinney, D.D.S.* 408

Some Points in the Correction of Irregularities, *By Dr. Russell Markwell* 415

Bleaching Enamel, - - *By Joseph Head, D.D.S.* 419

Professional Ethics, - - - *By C. N. Johnson* 421

EDITORIAL, - - - - - - - - - - 423

INDENTS, - - - - - - - - - - 425

